# Exploring the associations between neighbourhood food environment, household food insecurity and child weight-related outcomes in socio-economically and racially/ethnically diverse families

**DOI:** 10.1017/S1368980022002130

**Published:** 2022-10-10

**Authors:** Sarthak Agarwal, Angela R Fertig, Amanda C Trofholz, Allan D Tate, Jenna Robinson, Jerica M Berge

**Affiliations:** 1 Humphrey School of Public Affairs, University of Minnesota, 130 Hubert H. Humphrey Center, 301 19th Ave South, Minneapolis, MN 55455, USA; 2 Department of Family Medicine and Community Health, University of Minnesota Medical School, Minneapolis, MN, USA; 3 College of Public Health, University of Georgia, Athens, GA, USA

**Keywords:** Neighbourhood food environment, Household food security, Child diet quality, Home food availability

## Abstract

**Objective:**

To examine associations among neighbourhood food environments (NFE), household food insecurity (HFI) and child’s weight-related outcomes in a racially/ethnically diverse sample of US-born and immigrant/refugee families.

**Design:**

This cross-sectional, observational study involving individual and geographic-level data used multilevel models to estimate associations between neighbourhood food environment and child outcomes. Interactions between HFI and NFE were employed to determine whether HFI moderated the association between NFE and child outcomes and whether the associations differed for US-born *v*. immigrant/refugee groups.

**Setting:**

The sample resided in 367 census tracts in the Minneapolis/St. Paul, MN metropolitan area, and the data were collected in 2016–2019.

**Participants:**

The sample was from the *Family Matters* study of families (*n* 1296) with children from six racial/ethnic and immigrant/refugee groups (African American, Latino, Hmong, Native American, Somali/Ethiopian and White).

**Results:**

Living in a neighbourhood with low perceived access to affordable fresh fruits and vegetables was found to be associated with lower food security (*P* < 0·01), poorer child diet quality (*P* < 0·01) and reduced availability of a variety of fruits (*P* < 0·01), vegetables (*P* < 0·05) and whole grains in the home (*P* < 0·01). Moreover, residing in a food desert was found to be associated with a higher child BMI percentile if the child’s household was food insecure (*P* < 0·05). No differences in associations were found for immigrant/refugee groups.

**Conclusions:**

Poor NFE were associated with worse weight-related outcomes for children; the association with weight was more pronounced among children with HFI. Interventions aiming to improve child weight-related outcomes should consider both NFE and HFI.

Neighbourhood food environments (NFE) can contribute to public health outcomes including food security^(1,2)^ and optimal child weight^(3)^. Child weight may suffer if a neighbourhood lacks food outlets selling nutritious options at affordable prices by lowering energy intake that could lead to weight loss or lowering dietary quality that could lead to weight gain. Moreover, the associations between NFE and child weight may be more pronounced for children living in food-insecure households. According to the United Nations Food and Agriculture Organization, ‘Food security exists when all people, at all times, have physical, social and economic access to sufficient, safe and nutritious food which meets their dietary needs and food preferences for an active and healthy life’^(2)^. By definition, a neighbourhood with plentiful nutritious food retail and food service outlets increases two dimensions of food security: (1) availability, which ‘addresses whether or not food is actually present, including ... markets’ and (2) access, or ‘whether or not households and individuals have sufficient physical and economic access to that food’^(2)^. *In this study, we examined whether NFE were associated with household food insecurity (HFI) and child weight and weight-related outcomes, as well as whether HFI moderated the relationship between NFE and child weight and weight-related outcomes, and whether the association between NFE and outcomes differed for US-born v. immigrant/refugee groups*.

The definition of neighbourhood food environment varies substantially in the literature^(4)^. In this study, we conceptualise NFE as the distribution of food sources in a neighbourhood around one’s home, including the distance to food retail and service outlets, and the types of outlets present. While we expect variation in nutritional offerings within each outlet type^(5–7)^, we assume that supermarkets provide access to fresh produce, and fast-food restaurants and convenience stores offer mostly food choices of low nutritional value^(3,4)^. Fewer food outlets of any kind in the neighbourhood may result in low access to nutritious food and higher prices for all types of food, consequently leading to an inadequate quantity and/or quality of food consumed by families^(1)^.

Although the literature examining the association between NFE and childhood obesity and weight-related behaviours is large and growing, the evidence is mixed^(4,5,8)^. Some studies have concluded that distance to the nearest grocery store is unrelated to a child’s diet^(9)^, where others have found significant associations with greater fruit and vegetable consumption^(10)^ and lower child’s BMI^(10,11)^. Likewise, food deserts, defined by the US Department of Agriculture as ‘a low-income census tract with at least 500 people, or 33 percent of the population, living more than 1 mile (1·6 km) (urban areas) or more than 10 miles (16·1 km) (rural areas) from the nearest supermarket, supercenter, or large grocery store^(12)^,’ have been shown to be associated with weight status^(13)^, yet the entry of a new grocery store into a food desert was not found to improve obesity rates in the area^(14)^. Proximity to fast-food establishments has been found to be related to both child obesity and diet quality in some studies^(3)^; however, no association was found with weight-related behaviours in a recent systematic review^(3)^.

Similarly, while many studies have examined the relationship between household food insecurity, diet and weight, the results are often conflicting^(15)^. Food insecurity is theorised to lead to lower quality diets because families substitute away from nutritious foods (e.g. lean sources of protein, fruit and vegetables) to less nutritious energy-dense foods (e.g. hot dogs), which are cheaper per calorie^(16)^. At the same time, with very low food security, families may reduce the amount of energies consumed, especially among the adults in the households^(17)^. Thus, some studies find that food insecurity is associated with lower child diet quality^(18)^, and some find no association^(19)^; some studies find that food insecurity is associated with increases in child BMI^(20)^, while others find no association^(21)^.

There are several potential explanations for the mixed evidence of NFE. First, just as the association between food insecurity and outcomes varies across different types of families, NFE may matter more for some families than others^(8)^. Families experiencing food insecurity may switch to less nutritious foods or may be forced to cut energies more dramatically in poor NFE. Individuals who shop online or who have access to transportation may shop and dine outside of their neighbourhood^(22)^, making the neighbourhood food environment less important. Families who prefer to eat foods that are culturally relevant to their community may view their neighbourhood food environment as poor while families who prefer to eat ‘American’ foods may view the same neighbourhood as a food oasis^(4)^, or vice versa^(23)^.

Second, the definition and measurement of NFE vary widely from study to study^(4)^. Some studies used objective geographic measures of various food outlet locations^(10,11,24,25)^, but these measures fail to capture affordability, quality, cultural relevance or the customer experience (i.e. racism^(26)^ and safety^(27)^). Other studies used subjective perception measures that capture the nuances geographic measures cannot^(28,29)^, but may be subject to other issues, including the lack of awareness or experience with all the food outlets present in the neighbourhood, or response bias reflecting their justification for poor diet quality^(30)^.

This study aimed to contribute to the previous literature by examining the associations between neighbourhood food environment, household food insecurity and child weight-related outcomes using a rich data set of racially and ethnically diverse families with multiple measures (objective and subjective) of food environment. We expected that a poor neighbourhood food environment would be associated with less nutritious food present in the home and lower quality child diets for all families. For food-insecure families, we hypothesised that the effects of NFE might compound the effects of food insecurity such that the associations between NFE and child outcomes would be more pronounced. Finally, we hypothesised that the associations between NFE and outcomes might differ for immigrant/refugee groups (Latino, Hmong and Somali/Ethiopian) compared with US-born groups (White, African American and Native American) because of the cultural relevance of foods found in neighbourhoods.

## Methods

### Data

*Family Matters* is an incremental, mixed-methods study with two phases. Phase I was a cross-sectional, mixed-methods investigation of the home environment of low-income and racially/ethnically diverse families (*n* 150) with a 5–7-year-old child. Phase II, informed by findings from Phase I, is a longitudinal cohort study of low-income and racially/ethnically diverse families (*n* 1307) with a 5–9-year-old child. Details on Phase I are published elsewhere^(31)^. This cross-sectional study used baseline Phase II data collected via an online survey between November 2016 and November 2019.

All study protocols were approved by the Institutional Review Board at the University of Minnesota (1107S02666). All study materials (e.g. consent forms, survey questions) were translated into Hmong, Somali and Spanish. The translation was conducted by bilingual and bi-cultural staff members; study materials were first translated by one staff member and then reviewed by another staff member.

### Sample

Parent/child dyads were recruited for Phase II from primary care clinics in Minneapolis/St. Paul, Minnesota. The clinics identified 5–9-year-old children who had recently had a well-child visit, and therefore, a recent objective height and weight measurement. A letter was sent from the primary care clinic inviting the family to participate in the *Family Matters* study. Families were eligible to participate in the Phase II study if they met the following eligibility criteria: (1) the child was 5–9 years old; (2) the child’s BMI percentile was greater than 5; (3) the person completing the survey was the primary guardian and caregiver of the child; (4) the child lived with the respondent more than 50 % of the time and (5) the child was from one of the following racial/ethnic categories: African American, Latino, Hmong, Native American, Somali/Ethiopian or White. Of the 1307 families enrolled in the baseline Phase II study, 1296 families with complete information on all variables (eleven observations were missing income and were dropped) were analysed in this study. These 1296 families lived in 367 census tracts.

### Outcome measures

Four self-reported measures were used to capture household food availability. These measures were adapted from the Home Food Inventory^(32)^ so that participants could self-report each item. The Home Food Inventory was developed in the Twin Cities and has been validated for Latino and Somali families^(33)^. We asked the parent to count the number of different varieties of fruits and vegetables (separately) in the home and respond on a scale of 0–12 (where 0 = none, 1 = one to two, 4 = three to five, 7 = six to eight, 10 = nine to eleven and 12 = twelve or more). In addition, we created a variable that captures the fraction of available food products that would be classified as whole grain (e.g. bread, rice, noodles, crackers, etc.). The responses were classified on a scale of 0–2 (where 0 = no whole grain foods, 1 = mixture of whole and refined grain foods and 2 = all whole grain foods). Finally, we summed the number of different quick (i.e. pre-prepared, pre-packaged) foods present in the home from a list of the following eleven possibilities: hamburgers, frozen dinners, burritos, ramen noodles, macaroni and cheese, French fries, canned soup, pasta meals, chicken nuggets, corn dogs and noodle dishes. Higher numbers of fruits, vegetables and fraction whole grains indicate higher quality foods found in the home where higher numbers of quick foods indicate lower quality of foods found in the home^(34,35)^.

Frequency of fast-food meals per month (range 0–90) was collected using a shortened version of a fast-food consumption assessment from prior research^(36,37)^. The score was created from responses to the number of times the target child ate something from the following eight types of fast-food restaurants in the past month, including take-out and delivery: (1) coffee shops (e.g. Caribou, Starbucks and Dunn Bros.), (2) restaurants serving fried chicken (e.g. KFC, Popeyes), (3) Asian fast food (e.g. Panda Express, Leeann Chin), (4) pizza place, (5) sandwich or sub shop (e.g. Subway, Panera and Quiznos), (6) traditional ‘burger-and-fries’ fast-food restaurants (e.g. McDonald’s, Burger King, Wendy’s and Culver’s), (7) buffet (e.g. Old Country Buffet) and (8) Mexican fast-food restaurants (e.g. Taco Bell, Taco John’s and Chipotle)^(37)^. Response options for each food outlet included 0 = never/rarely, 2 = 1–2 times per month, 6 = 1–2 times per week, 15 = 3–4 times per week, 23 = 5–6 times per week and 30 = 1+ times per day. The responses were summed and truncated at ninety fast-food meals per month because of the implausibility of eating more than three fast-food meals per day on average (twenty responses (1·5 %) were truncated). A higher number of fast-food meals indicate lower diet quality^(38)^.

Child’s dietary intake quality was measured using a child diet quality scale (possible range 0–100) based on an adapted version of the Children’s Eating Habits Questionnaire that had been tested in a validity sub-study in Phase I of the *Family Matters* study^(39)^. This adapted measure is like a FFQ. The parent was asked to report on the child’s dietary intake within the past 4 weeks. To capture overall dietary intake quality, staff dietitians created a score using the Healthy Eating Index-2015 as a guide. The Healthy Eating Index-2015 has been shown to be reliable and valid in assessing diet quality^(40)^. Similar to the Healthy Eating Index-2015, the dietary quality score created using the *Family Matters* study gives participants points for consuming healthy food categories (e.g. dark green vegetables) and for not consuming food categories that should be eaten in moderation (e.g. Na)^(39)^. The points for each category were then summed to create an overall dietary quality score where higher scores indicate a higher diet quality.

Child BMI percentile (pBMI) (range 5–99·9) was calculated using height and weight measurements taken from the child’s last well check visit at the primary care clinic via the electronic health record. The families were recruited from multiple primary care clinics across the Twin Cities. The percentiles were based on the 2000 US Centers for Disease Control and Prevention Growth Reference by gender and age in months. We treated child pBMI as a continuous variable, where higher values indicate moving closer to the thresholds of 85 for overweight and 95 for obese.

A household food-insecurity scale (range 0–6) was created by summing the parent’s responses to six questions from the USDA’s six-item questionnaire (0 = high food security and 6 = very low food security)^(41,42)^. This scale was also dichotomised into ‘food insecure’ (if the scale was two or more) and ‘food secure’ (if the scale was 0 or 1) households.

### Neighbourhood food environment measures

The following three measures were used to capture the neighbourhood food environment. First, a low perceived access to fruits/vegetables score was created (range 0–12) by summing responses to three Likert questions (0 = strongly agree to 4 = strongly disagree): if the fruits and/or vegetables available at stores in the neighbourhood are (1) easily available; (2) affordable and (3) high quality. This instrument has been used in a prior study on a similar sample from the Twin Cities assessing perceptions of the food environment^(43,44)^. A higher score indicates lower perceived access to fruits/vegetables. Second, the proximity to unhealthy food retail options score (range 0–8) is a sum of the responses about walking distances (without children) to (1) convenience stores and (2) fast-food restaurants. The response options for distance to a food retail outlet were 0 = more than 30 min, 1 = 21–30 min, 2 = 11–20 min, 3 = 5–10 min and 4 =< 5 min. These questions are part of a larger assessment^(45)^ designed to capture neighbourhood walkability and were repurposed in this study to capture proximity. A higher score indicates closer proximity to convenience stores and fast-food restaurants. Finally, we matched each household’s address to census tract-level data from the US Department of Agriculture on whether a census tract is a food desert, an indicator variable defined above^(12)^.

### Covariates

We included the following covariates to adjust for socioeconomic characteristics of the census tract and the individual household. The area deprivation index is a census tract-level variable that is intended to capture neighbourhood disadvantage generally. The measure is created using data from the American Community Survey on income, education, employment and housing quality^(46)^. This covariate allows us to estimate the associations with the food environment independent of the economic condition of the neighbourhood. At the individual level, we adjusted for the child’s race/ethnicity and gender, the primary caregiver’s education and the household’s income and public assistance receipt. Public assistance receipt is intended to capture additional resources coming into the household through receipt of two government cash benefit programs (Temporary Aid for Needy Families, which is called Minnesota’s Family Investment Program in Minnesota, and Supplemental Security Income), and participation in three major US food programs (Supplemental Nutrition Assistance Program, the Special Supplemental Nutrition Program for Women, Infants and Children, and the National School Breakfast or Lunch Programs), which have been shown to affect food insecurity and child weight-related outcomes^(47)^. We also controlled for household composition (i.e. the number of adults and children). Because the neighbourhood food environment may be less important to households that shop for groceries online or those who have access to a car, we controlled for whether the respondent reported ever having shopped for groceries online and whether they usually travel to grocery stores via their own, family or a friend’s car. Finally, we adjusted for food acculturation and time in the USA because the NFE may be perceived differently among immigrant/refugee families who eat mostly cultural foods or have not lived in the USA for very long. Food acculturation was captured with one question that asks parent respondents whether ‘the food I eat at home is from (a) the US, (b) the country that my family is from, (c) both or (d) neither.’ We created an indicator for eating American food at home if parents reported a or c. Time in the USA was captured through two questions: one about being born in the USA and one about how long one has lived in the USA if they reported not being born in the USA. Using these questions, parents were divided into three groups: born in the USA, not born in the USA but lived in the USA 10+ years, and not born in the USA and lived in the USA < 10 years.

### Statistical analysis

To describe how NFE differ by race/ethnicity, income, food acculturation and time in the USA, we used *t* tests to compare the three neighbourhood food environment variables (low perceived access to fruits/vegetables, proximity to unhealthy food retail options and food desert) by race/ethnicity, income, food acculturation and time in the USA. The comparisons by food acculturation and time in the USA were restricted to the subsample of immigrant groups (Latino, Hmong and Somali/Ethiopian). To estimate the association between the neighbourhood food environment and outcomes, we used multilevel linear models because we have both individual and census tract-level data (food desert and area deprivation index). We treated all eight outcomes (number of fruits, number of vegetables, fraction whole grain, number of quick foods, frequency of fast-food meals per month, child diet quality scale, child pBMI and the food security scale) as continuous random variables. To determine whether the associations vary by food security status, we ran multilevel regressions with interactions between food security status and the three neighbourhood food environment measures. For the outcomes where any of the three interaction terms were significant, we ran multilevel regressions separately for food-insecure households (*n* 385) and food-secure households (*n* 911). To determine whether the associations vary for US-born groups *v*. immigrant/refugee groups, we ran multilevel regressions with interactions between immigrant group status and the three neighbourhood food environment measures. Robust standard errors were used, and no multicollinearity issues were found. In particular, the correlations across the three food environment measures were low (see Appendix Table A). All regressions were adjusted for the covariates listed above. Statistical analyses were performed in Stata 17·0 SE.

## Results

Table 1 describes the characteristics of the families analysed (*n* 1296). The average family in the sample had two adults and three children. All but 14 % of the parents had a high school degree or more. Regarding public assistance, 35 % received Supplemental Nutrition Assistance Program benefits, and almost half of the sample was receiving Special Supplemental Nutrition Program for Women, Infants and Children and/or free or reduced-price breakfasts or lunches at school. Regarding income, 30 % of families made < $20 000 and an additional 25 % made between $20 000 and $34 999. Approximately 91 % of the sample had access to a car and only 10 % had ever shopped online for groceries. Eighty percent of Latino, Hmong and Somali/Ethiopian families reported eating some American foods at home, and 82 % of Latino, Hmong and Somali/Ethiopian parent respondents either were born in the USA or had lived in the USA for more than 10 years.

### Neighbourhood food environments by race/ethnicity and income

Perceived access to fruits and vegetables was significantly lower (*P* < 0·01) for all income groups relative to those with household incomes above $75 000 (top panel of Table 2). Similarly, it was significantly lower (*P* < 0·01) for all racial/ethnic groups when compared with White households (middle panel of Table 2). Proximity to unhealthy food retail options was significantly closer for the two lowest income categories (*P* < 0·05) but varied by race/ethnicity. The percentage of households living in a food desert was significantly higher (*P* < 0·01) for the middle-income categories (between $20 000 and $74 999) when compared with the highest income category. With respect to race/ethnicity, this percentage was significantly higher for African American (*P* < 0·01), Latino (*P* < 0·05) and Hmong (*P* < 0·01) households when compared with White households. We observed no significant differences in NFE among Latino, Hmong and Somali/Ethiopian families by whether they eat American foods at home. However, Latino, Hmong and Somali/Ethiopian parent respondents who have lived in the USA for < 10 years are significantly less likely to live in a food desert than Latino, Hmong and Somali/Ethiopian respondents who were born in the USA (9·7 % *v*. 28·3 %, *P* < 0·01), but their perception of their access to fruits and vegetables and their proximity to unhealthy food retail options were the same.

### Associations between neighbourhood food environment and weight-related outcomes

The regression results (Table 3) show that low perceived access to fruits and vegetables was significantly associated with less healthful home food availability – specifically, fewer varieties of fruits (*P* < 0·01) and vegetables (*P* < 0·05) and a lower fraction of whole grains (*P* < 0·01) – as well as poorer overall child diet quality (*P* < 0·01). In terms of magnitude, a family whose perceived access score was 12 (extremely low access to affordable fruits and vegetables) had on average 1·4 (=12 × –0·115) fewer fruit varieties at home, and a child diet quality score 4·2 units (=12 × –0·348) lower, than a family with the lowest score (0 = high access). Proximity to unhealthy food outlets was associated not only with a lower fraction of whole grains (*P* < 0·05) but also fewer varieties of quick foods (*P* < 0·05) available in the home. Residing in a food desert was not significantly associated with home food availability, fast food frequency or child diet quality. The pBMI of the child was unrelated to any of the neighbourhood food environment measures.

### Associations between neighbourhood food environment and food insecurity

Low perceived access to fruits and vegetables (Table 3, last row) was significantly associated with food insecurity (*P* < 0·01). A family whose low perceived access score was 12 had an average food insecurity score that was 1·3 points (=12 × 0·110) higher (indicating greater food insecurity) than a family with the lowest score, holding all else constant. Similarly, if households were in a food desert, they were more likely to be food insecure (*P* < 0·05). There was no significant association, however, between food insecurity and a household’s proximity to unhealthy food retail options.

### Does household food security status moderate the associations between food environment and outcomes?

Food security status was significantly related to the association between food desert status and fraction whole grains (*P* < 0·05) and child pBMI (*P* < 0·001) (Table 4). For all the other outcomes, the association with any of three NFE was not significantly different by food security status. Living in a food desert was significantly associated with having a higher fraction of whole grains in their home (*P* < 0·05) if the household was food secure, but not if they were food insecure. In contrast, residing in a food desert was found to be associated with a higher child BMI percentile if the child’s household was food insecure (*P* < 0·05), but not if the child’s household was food secure.

### Do the associations between food environment and outcomes vary for US-born groups v. immigrant groups?

For all the outcomes, the association with any of three NFE was not significantly different for immigrant/refugee groups (Latino, Hmong and Somali/Ethiopian) *v*. US-born groups (White, African American and Native American). As a result, we do not report analyses separated by this status.

## Discussion

Our results showed that families from lower income and non-White households were significantly more likely to live in poor NFE. This is consistent with previous studies^(30)^. Our hypotheses about the relationship between poor NFE and less nutritious food availability in the home and low child dietary quality were supported and cohere with other research^(29)^. In addition, consistent with Ma (2016)^(48)^, we found that families who perceived lower access to fruits and vegetables were significantly more likely to be food insecure. This finding is consistent with the idea that a neighbourhood that lacks plentiful nutritious food retail and food service outlets decreases the availability and accessibility dimensions of food security.

For food-insecure families, we hypothesised that the effects of NFE might be more pronounced than for food-secure families because NFE may compound the effects of food insecurity. While the results indicated that the associations between poor NFE and the presence of lower quality foods and diets were similar for both food-secure and food-insecure families, they also indicated that food deserts were associated with a higher pBMI among children living in food-insecure households, but not those living in food-secure households. These findings suggest that, for children in food-insecure households, the association between NFE and the quality of foods consumed dominates any quantity effect (i.e. lower energy intake). Moreover, because food insecurity involves having enough food some days but not others, the snapshots of home food inventory and child diet that we observed may not reflect the cumulative effects of a poor NFE on the health of children living in food-insecure households as well as percentile BMI.

We hypothesised that cultural differences in food preferences and their perceptions of food outlets may have also affected the study results. However, we did not observe significant differences in the NFE among Latino, Hmong and Somali/Ethiopian families by their acculturation to American food. We observed that Latino, Hmong and Somali/Ethiopian parents who were born in the USA are more likely to live in a food desert than more recent immigrants, but this correlation is likely driven by racial/ethnic differences. That is, Hmong immigration to Minnesota occurred in the 1970s, thus only 4 % of Hmong parents in our sample have lived in the USA < 10 years. In contrast, 37 % of Latino parents and 22 % of Somali/Ethiopian parents in our sample are recent immigrants (< 10 years) to the USA. Hmong families live primarily on the eastern part of St. Paul where grocery stores are more spread out than in areas closer to downtown Minneapolis or St. Paul, where many Somali/Ethiopian families reside. Moreover, interactions indicated that the associations between NFE and outcomes were not significantly different for the White, African American and Native American families compared with the Latino, Hmong and Somali/Ethiopian families.

The results of this study highlight numerous shortcomings in our ability to holistically measure NFE. Our objective measure, food desert status, was related to weight status (as has been found previously^(11)^) and food insecurity, but not related to household food availability or diet quality. In contrast, our subjective measure, low perceived access to fruits/vegetables, was related to household food availability, diet quality (consistent with some^(29)^ but not all studies^(28)^) and food insecurity, but not related to weight. As discussed above, objective measures of the presence of food outlets do not capture the quality of offerings available in stores^(26)^ and restaurants, which may explain why they were unrelated to measures of food quality in this study. Perceptions of access may more accurately capture the customer experience inside stores and restaurants including racism^(26)^ and safety^(27)^ and the quality and cultural relevance of foods available. Findings from Table 2 support this argument; even though relatively few Somali/Ethiopian and Native American families live in food deserts, the perception of their access to fruits and vegetables is significantly lower than White families, who live in food deserts at the same rate. Finally, this study focuses on the area surrounding the family home, but areas surrounding schools and work may also be relevant depending on the family^(9,49)^.

In addition to these measurement issues, this study has other limitations. First, the study was cross sectional. Further research is needed to trace the effects of any changes in food environment over time using a longitudinal study design. Second, families in the sample may have selected their neighbourhoods based on its food environment, which implies that improving the NFE would have no effect on health outcomes. However, because of residential racial segregation in the Twin Cities^(50)^, we argue that selection bias due to neighbourhood choice was low for non-white families, which comprise 82 % of the full sample. Research on racial covenants and redlining in Minneapolis/St. Paul document that home sales to minorities were blocked in the early 1900s and that these policies established patterns of residential racial segregation in the Twin Cities that persist today^(50)^. Third, some parents may have had difficulty answering questions about specific foods or restaurants if their knowledge of food and nutrition or their exposure to US foods was low.

## Conclusion

This study uses a rich set of subjective and objective measures of neighbourhood food environment and outcomes, as well as a large, racially/ethnically diverse and immigrant/refugee sample to examine the associations among neighbourhood food environment, household food insecurity and child’s weight and weight-related outcomes. Poor NFE were found to be associated with worse weight-related outcomes for children, and the association with weight was more pronounced among children living in food-insecure households. Policies and interventions aiming to improve child weight-related outcomes and food insecurity may want to consider the neighbourhood food environment and aim to remove environmental barriers to food security and healthy eating. Increasing the availability and accessibility of food outlets that provide nutritious, culturally relevant foods in a safe and inclusive manner may contribute to reducing disparities in childhood obesity.

## Figures and Tables

**Table 1:** Descriptive statistics of *Family Matters* study participants (*n* 1296)

	Mean/Percentage	SD

Area Deprivation Index* (range 49–177)	117·3	29·5
Shop online for groceries ever	0·10	0·30
Use own or friend/family car for grocery shopping	0·91	0·29
Number of adults in home (range 1–6)	2·01	0·92
Number of children in home (range 1–6)	2·89	1·37
Child is female	0·49	0·50
Primary caregiver’s education		
Less than High School	0·14	0·35
High School graduate	0·40	0·49
Some college	0·32	0·46
College Graduate	0·15	0·35
Public assistance receipt		
SNAP	0·35	0·48
WIC and/or free school breakfast and/or lunch	0·48	0·50
Welfare	0·14	0·35
SSI	0·09	0·29
Household income		
< $20 000	0·30	0·46
$20 000–$34 999	0·25	0·43
$35 000–$49 999	0·16	0·36
$50 000–$74 999	0·11	0·31
$75 000+	0·18	0·39
Racial/Ethnic group		
White	0·18	0·39
African American	0·21	0·41
Latino	0·16	0·37
Hmong	0·17	0·38
Somali/Ethiopian	0·10	0·30
Native American	0·16	0·37
Latino, Hmong, Somali/Ethiopian groups only** (*n* 570)		
Eat American foods at home	0·80	0·40
Born in US	0·27	0·44
Lived in US 10+ years	0·55	0·50
Lived in US < 10 years	0·18	0·39

* Area Deprivation Index captures socio-economic disadvantage of a Census tract based on income, education, employment and housing quality of residents.

** Eat American foods at home, born in the USA and how long someone has lived in the USA are asked of all respondents; we report statistics only for Latino, Hmong, Somali/Ethiopian groups because the responses for White, African American and Native American respondents are almost universally Eat American foods at home and Born in US.

**Table 2:** Variation in neighbourhood’s food environment by household income, race/ethnicity, food acculturation and time in the USA

	# households	# census tracts	Low perceived access to fruits/ vegetables score (range 0–12)	Proximity to unhealthy food retail options score (range 0–8)	Census tract is food desert (%)

Full sample	1296	367	3·23	4·16	16·6
Household income					
< $20 000	393	163	3·88**	4·32*	14·5
$20 000–$34 999	323	156	3·36**	4·36*	17·3**
$35 000–$49 999	203	113	3·28**	4·17	20·2**
$50 000–$74 999	143	104	3·01**	3·58	26·6**
$75 000+ (ref)	234	146	2·03	3·97	9·8
Racial/Ethnic group					
African American	278	152	3·36**	4·45*	20·5**
Latino	212	116	3·67**	3·78	18·4*
Hmong	226	79	3·13**	3·67*	27·4**
Somali/Ethiopian	132	64	3·12**	4·33	7·6
Native American	210	86	4·12**	4·68**	11·0
White (ref)	238	152	2·04	4·07	10·1
Food acculturation (Latino, Hmong, Somali/Ethiopian only)					
Eat American Foods at Home					
No	114	64	3·62	4·14	14·0
Yes (ref)	456	178	3·25	3·80	20·8
Time in the USA (Latino, Hmong, Somali/Ethiopian only)					
Lived in US < 10	103	55	3·48	4·14	9·7**
Lived in US 10+	315	140	3·33	3·82	18·4*
Born in US (ref)	152	83	3·21	3·78	28·3

* *P* < 0·05.

** *P* < 0·01.

Significance tests are relative to the last group listed in each panel. Example interpretations: Sample families with household incomes below $20 000 (*n* 393) live in 163 census tracts in the Twin Cities and have an average low perceived access to fruit/vegetable score of 3·88, which is statistically significantly (*P* < 0·01) above the score for sample families with household incomes above $75 000 (2·03, *n* 234, reference group). African American families in the sample (*n* 278) live in 152 census tracts in the Twin Cities and have an average low perceived access to fruit/vegetable score of 3·36, which is statistically significantly (*P* < 0·01) above the score for White families in the sample (2·04, *n* 238, reference group). Latino, Hmong and Somali/Ethiopian families who report that at home they do not eat American foods (*n* 114) live in 64 census tracts and have an average low perceived access to fruit/vegetable score of 3·62, which is not statistically significantly different from the score for Latino, Hmong and Somali/Ethiopian families who report that at home they eat American foods (3·25, *n* 456, reference group). Latino, Hmong and Somali/Ethiopian parent respondents who have lived in the USA for < 10 years (*n* 103) live in 55 census tracts and 9·7 % live in a food desert, which is significantly less (*P* < 0·01) than Latino, Hmong and Somali/Ethiopian respondents who were born in the USA (28·3 %, *n* 152, reference group).

**Table 3:** Associations between household’s food security and food availability, child’s BMI percentile and diet quality and neighbourhood food environment (*n* 1296 from 367 census tracts)

	Mean	SD	Low perceived access to fruits/vegetables score (range 0–12)		Proximity to unhealthy food retail options score (range 0–8)		Census tract is food desert (yes/no)	
			Coef.	95 % CI	Coef.	95% CI	Coef.	95 % CI

# fruits (range 0–12)	4.98	2.75	−0.115**	−0.172, −0.057	−0.024	−0.103, 0.055	−0.284	−0.692, 0.124
# vegetables (range 0–12)	6.06	3.00	−0.073*	−0.134, −0.013	0.011	−0.084, 0.106	−0.149	−0.555, 0.256
Fraction whole grain (0, 1 or 2)	1.17	0.64	−0.016**	−0.029, −0.004	−0.018*	−0.035, −0.000	0.035	−0.069, 0.140
# quick foods (range 0–11)	4.62	2.98	0.045	−0.023, 0.112	−0.097**	−0.162, −0.032	0.150	−0.280, 0.579
# fast food/month (range 0–90)	11.25	15.02	−0.007	−0.362, 0.349	0.039	−0.389, 0.467	0.647	−1.628 −2.921
Child diet quality scale (range 0–85)	57.33	10.31	−0.348**	−0.579, −0.118	0.139	−0.174, 0.453	−0.524	−1.830, 0.781
Child pBMI (range 5–99.9)	66.27	28.30	0.131	−0.499, 0.762	0.028	−0.815, 0.871	0.315	−3.492, 4.122
Food-insecurity scale (range 0–6)	1.18	1.82	0.110**	0.075, 0.144	−0.013	−0.061, 0.034	0.350*	0.050, 0.650

pBMI, BMI percentile.

* *P* < 0.05.

** *P* < 0.01.

Results from multilevel linear regressions shown, where the food desert variable is at the census tract level. Each row represents one regression with the variable listed on the left as the outcome. All regressions are adjusted for area deprivation index, online shopping experience, shopping by car, number of adults, number of children, child’s gender, primary caregiver’s education, public assistance receipt, household income, race/ethnicity, eating American foods at home and time in the USA. Example interpretation: After adjusting for covariates, a higher score of low perceived access to fruits and vegetables is associated with a lower variety of fruits available in the home (coef = −0.115, *P *< 0.01), but proximity to unhealthy food retail options and residing in a food desertare not statistically significantly associated with the number of fruits available in the home.

**Table 4:** Associations between household’s food availability, child’s BMI percentile and diet quality and neighbourhood food environment, by household food security status

			Low perceived access to fruits/vegetables scale (range 0–12)		Proximity to unhealthy food retail options score (range 0–8)		Census tract is food desert (Yes/No)	
	Mean	SD	Coef.	95 % CI	Coef.	95 % CI	Coef.	95 % CI

	Food-insecure households (*n* 385 from 170 census tracts)							
Fraction whole grain (0, 1 or 2)	1.08	0.69	−0.011	−0.034, 0.013	−0.001	−0.033, 0.032	−0.108	−0.282, 0.066
Child pBMI (range 5–99.9)	71.89	26.32	−0.497	−1.503, 0.509	0.482	−0.912, 1.876	5.723*	0.560, 10.887
	Food-secure households (*n* 911 in 319 census tracts)							
Fraction whole grain (0, 1 or 2)	1.21	0.62	−0.018*	−0.033, −0.002	−0.029**	−0.049, −0.009	0.122*	0.009, 0.235
Child pBMI (range 5–99.9)	63.89	28.78	0.350	−0.440, 1.139	−0.096	−1.113, 0.921	−3.898	−8.803, 1.008

NFE, neighbourhood food environment; pBMI, BMI percentile.

* *P* < 0–05.

** *P* < 0–01.

Results from multilevel linear regressions shown, where the food desert variable is at the census tract level. Each row represents one regression with the variable listed on the left as the outcome. All regressions are adjusted for area deprivation index, online shopping experience, shopping by car, number of adults, number of children, child’s gender, primary caregiver’s education, public assistance receipt, household income, race/ethnicity, eating American foods at home and time in the USA. Only those outcomes where an interaction analysis indicated that the association between NFE and the outcomes differed significantly by food security status are reported. Example interpretation: After adjusting for covariates, living in a food desert is significantly associated with a 5.723 higher child pBMI (*P* < 0.05) for food-insecure households but not for food-secure households.
